# Combined Activity of DCL2 and DCL3 Is Crucial in the Defense against *Potato Spindle Tuber Viroid*

**DOI:** 10.1371/journal.ppat.1005936

**Published:** 2016-10-12

**Authors:** Konstantina Katsarou, Eleni Mavrothalassiti, Wannes Dermauw, Thomas Van Leeuwen, Kriton Kalantidis

**Affiliations:** 1 Institute of Molecular Biology and Biotechnology, Foundation for Research and Technology-Hellas, Heraklion, Greece; 2 Department of Biology, University of Crete, Heraklion, Greece; 3 Laboratory of Agrozoology, Department of Crop Protection, Faculty of Bioscience Engineering, Ghent University, Belgium; 4 Institute for Biodiversity and Ecosystem Dynamics, University of Amsterdam, The Netherlands; University of California Riverside, UNITED STATES

## Abstract

Viroids are self replicating non-coding RNAs capable of infecting a wide range of plant hosts. They do not encode any proteins, thus the mechanism by which they escape plant defenses remains unclear. RNAi silencing is a major defense mechanism against virus infections, with the four DCL proteins being principal components of the pathway. We have used *Nicotiana benthamiana* as a model to study *Potato spindle tuber viroid* infection. This viroid is a member of the *Pospiviroidae* family and replicates in the nucleus via an asymmetric rolling circle mechanism. We have created knock-down plants for all four DCL genes and their combinations. Previously, we showed that DCL4 has a positive effect on PSTVd infectivity since viroid levels drop when DCL4 is suppressed. Here, we show that PSTVd levels remain decreased throughout infection in DCL4 knockdown plants, and that simultaneous knockdown of DCL1, DCL2 or DCL3 together with DCL4 cannot reverse this effect. Through infection of plants suppressed for multiple DCLs we further show that a combined suppression of DCL2 and DCL3 has a major effect in succumbing plant antiviral defense. Based on our results, we further suggest that Pospoviroids may have evolved to be primarily processed by DCL4 as it seems to be a DCL protein with less detrimental effects on viroid infectivity. These findings pave the way to delineate the complexity of the relationship between viroids and plant RNA silencing response.

## Author Summary

Viroids consist of a peculiar type of highly structured small circular RNAs, capable of infecting crop plants and ornamentals. They do not encode any protein, yet they manage to replicate, move through the plant and often cause severe symptoms. In order to achieve this, viroids hijack plant cellular machinery. In addition, they manage to overcome plant RNAi response, which is the major antiviral defense mechanism of plants. DCL proteins have a central role in the RNAi pathway. We have used *Nicotiana benthamiana* plants, an experimental host of some viroids, and produced plants suppressed for DCL proteins (individually or in combination). By infecting DCL knockdown plants with *Potato spindle tuber viroid* we were able to identify which DCL proteins are mainly involved in the antiviroid response. We found that it is the combined activity of DCL2 and DCL3 pathways which most potently suppress viroid infectivity. In contrast, DCL4, the main antiviral DCL, seemed to obscure the DCL2-DCL3 effect on viroid infectivity. This lead us to the hypothesis that viroids may have evolved to be primarily processed by DCL4, as it seems to be the DCL protein with less detrimental effects on viroid infectivity. Our findings may aid understanding the complex interaction of viroids with the plant defense machinery.

## Introduction

Viroids are infectious, naked circular RNAs sized from 246 to 401 nucleotides (nt), capable of infecting a wide range of hosts, causing important economic loses [1]. They are divided into two families, *Pospiviroidae* and *Avsunviroidae* [1–3]. *Potato spindle tuber viroid* (PSTVd), a type species of the *Pospiviroidae* family, has a rod-like secondary structure with five distinct domains, and replicates in the nucleus through an asymmetric rolling circle mechanism using RNA polymerase II (RNAPII) [1,4–6]. It is known to infect crop plants of the *Solanaceae* family such as tomato and potato, as well as some ornamental plants of the *Scrophulariaceae* and *Asteracae* family, but does not infect the plant model *Arabidopsis thaliana* systemically.

Since viroids do not encode any protein they rely on plant available resources and / or mechanisms for their infectivity. One of the mechanisms they have been proposed to exploit is RNA interference (RNAi), especially because of their particular double stranded RNA structures (dsRNA) [4,5]. RNAi is an epigenetic process that regulates gene expression at transcriptional and post-transcriptional level and is one of the major plant defense mechanisms against viruses [7,8]. In plants, endogenous or exogenous double stranded or aberrant RNAs are recognized by specific proteins named Dicer-Like (DCL), and digested into double stranded small interfering RNAs (siRNAs) of 21 to 24nt [9]. These siRNAs are then incorporated into the RNA induced silencing complex (RISC) whose major components are Argonaute proteins (AGO), after which one of the strands is removed [9]. These complexes can recognize single stranded RNAs with good complementarity driving their degradation. In addition, they can serve as primers for the synthesis of dsRNA by an RNA-dependent RNA polymerase (RdRP), which leads to the production of secondary siRNAs thus amplifying the suppressive phenomenon [9].

In *Nicotiana benthamiana*, four DCL proteins have been described, with close homology with the ones in *A*. *thaliana* [10]. All four proteins contain six specific domains: DEAD-Helicase, Helicase C, DSRD, PAZ, RNAseIII and dsRBD [11]. Each DCL holds a specific and rather defined role. DCL1 is involved in the miRNA (micro-RNAs) biogenesis pathway and produces 21nt small RNAs from precursors named primary miRNA (pri-miRNA) (reviewed in [12]). DCL1 has also been proposed to affect DNA methylation, contribute to the silencing of certain transposons and finally facilitate the biogenesis of DNA virus siRNAs by other DCLs [13,14]. DCL2 generates 22nt long siRNAs of exogenous origin and 22nt natural antisense siRNAs [15,16]. DCL2 is involved in the production of secondary siRNAs which trigger the phenomenon of transitivity [17,18]. In addition, a role in the antiviral defense together with DCL4 is well established [15,19,20]. The main reported role of DCL3 is to form 24nt-mers related to RNA-directed DNA methylation, however its involvement in the production of 23 to 25nt long-miRNAs generated by miRNAs precursors has also been shown [21]. DCL4 is in charge of processing endogenous 21nt trans-acting RNAs (tasi-RNAs) but also of specific miRNAs in *A*. *thaliana* such as mir822 and mir839 [22,23]. Recently, a role in transcription termination was also described [24,25]. Nevertheless, the principal role of DCL4 is considered to be the antiviral capacity of this protein [19,20]. Of particular note is that even though the role of each DCL seem to be rather specific, redundancy between these pathways has been proposed [13,19,20,26–28].

RNAs are then incorporated into AGO containing complexes. Ten different AGO proteins are found in *A*. *thaliana* and seven in *N*. *benthamiana* [10,29,30]. In *A*. *thaliana*, AGO1 loads DCL1 products while AGO2 loads trans-acting RNAs and repeat associated RNAs [29]. Both AGO1 and AGO2 are major plant antiviral proteins depending on the virus [30]. For instance, AGO1 is involved in the defense against *Turnip crinkle virus* (TCV-Family: *Tombusviridae*, Genus: *Carmovirus*) and C*ucumber mosaic virus* (CMV-Family: *Bromoviridae*, Genus: *Cucumovirus*) whereas AGO2 in the defense of *Tobacco rattle virus* (TRV-Family: *Virgaviridae*, Genus: *Tobravirus*) and *Turnip mosaic virus* (Family: *Potyviridae*, Genus: *Potyvirus*) [31–34]. An additional minor role in plant defense has been attributed to AGO5, AGO7 and AGO10 [30,34]. 24nt are loaded into AGO4 and possibly AGO6, and are involved in RNA-directed DNA methylation [29]. AGO4 has also been involved in antiviral defense against RNA or DNA viruses, but most of its actions are related to perturbations of DNA methylation processes [30]. It is to note that recent findings propose an interaction of AGO4 with RNAPII in the plant nucleus [35].

The relation of viroids to the gene silencing mechanisms has been rather puzzling since contradictory reports about a positive or a negative role of gene silencing, have been published. In 2001, two independent studies suggested that viroids are targeted by plant defense, since small RNAs derived from the viroid genome (vd-siRNAs) could be detected in the host plants [36,37]. However in 2003, Chang *et al*. demonstrated *in vitro* that human Dicer was unable to cleave PSTVd [38]. A year later Wang *et al*. showed that tomato plants expressing RNA hairpins against PSTVd presented phenotypic similarities to infected plants suggesting an important role of vd-siRNAs in targeting endogenous transcripts but not the PSTVd genome [39]. This was further supported by two independent studies in 2007, where it was suggested that viroid genome is not targeted upon infection [40,41]. In contrast, a research study from Schmind *et al*. (2009) proposed that RNAi is able to counteract PSTVd infection [42]. The authors of this work showed that transgenic plants expressing a hairpin against PSTVd cannot be infected by the viroid which suggests that the viroid is targeted by the produced vd-siRNAs [42]. Targeting of specific endogenous genes by vd-siRNAs is now accepted; however, the question of if and how the targeting of viroid genome is achieved is still under investigation [43,44].

On the other hand, there were studies showing an involvement of specific RNAi components in the defense against viroids. RDR6 was shown to delay PSTVd infection, since plants with decreased RDR6 levels presented increased viroid titer compared to WT plants [45]. This increase was only visible in early time points, showing that RDR6 suppression is important mainly in the initial steps of PSTVd infection [45]. In 2013, we showed that DCL4 protein is somehow involved in the infection caused by PSTVd [46]. Specifically, knocked-down *N*. *benthamiana* plants for each and every DCL protein were produced and infected with the viroid. At 3 weeks post infection (wpi) plants with decreased DCL4 levels (DCL4i) repeatedly presented lower viroid levels compared to WT plants. This observation was in striking contrast to what is observed in viral infections, since when this protein is decreased or absent, a viral enhancement is usually observed. This indicated that the interplay between the silencing pathway and viroids does not follow current theory for anti-viral responses [13,19,20]. A recent publication by Minoia and colleagues showed that different AGO proteins bind vd-siRNAs [47]. AGO1, AGO2 and AGO3 bind 21 and 22nt vd-siRNAs, whereas AGO4, AGO5 and AGO9 additionally bind 24nt vd-siRNAs [47]. In this work they also showed that overexpression of *A*. *thaliana* AGO1, AGO2, AGO4 and AGO5 in infected plants drives a decrease in PSTVd levels, suggesting a targeting of the viroid by vd-siRNAs [47]. Lastly, advances of deep sequencing technologies showed that vd-siRNAs are of both polarities and are produced from the entire viroid genome [48–50].

In the present work, we attempt to revisit the complex interplay that viroids have with the RNAi silencing machinery. We have focused on detailing the effect of individual DCL genes and their combinations on PSTVd infectivity. We provide, for the first time, evidence that it is the combined activity of the DCL2 and DCL3 pathways that are required to efficiently suppress PSTVd. In addition, we suggest that viroids may have evolved to be primarily targeted by DCL4, a DCL protein with less detrimental effects on its infectivity, to avoid the more potent anti-viroid effect of the DCL2-DCL3 pathways.

## Results

### PSTVd infection in DCL4i plants

In order to further investigate our previous observation of the reduction of PSTVd levels in DCL4i plants [46], we first tested potential limitations of the experimental design that could interfere with the observed phenomenon. Firstly, we asked whether the hairpin itself is likely to interfere with the detected reduction in viroid levels by directly targeting the viroid. We used a blastn approach and evaluated whether artificially generated 21nt siRNAs derived from the DCL4.9i hairpin (DCL4hp) sequence (336 sequences in total; S1 Table) might target the PSTVd^NB^ genome. Only seven blastn-hits had an alignment length of more than 15 nucleotides (DCL4_hairpin_123-DCL4_hairpin_129; S1 Table) and all seven blastn hits had mismatches in the siRNA guide strand seed region. It is known that mismatches at this region perturb their function. In addition, leaves of PSTVd^NB^ infected plants were agroinfiltrated in one half with GFP and the other half with DCL4hp for three days, time sufficient for the hairpin to be expressed but not for the targeted protein to be significantly reduced. No significant difference in viroid levels were observed in the presence of DCL4hp (S1 Fig), suggesting that the hairpin does not directly target the viroid sequence.

Next, we used tissue print hybridization to detect possible effects of DCL4 suppression on the spatial distribution of PSTVd. No significant differences were observed in the infection of individual leaves between WT and DCL4i plants (S2 Fig)

We have also investigated whether the agroinfiltration method used for infection could influence our results. For this reason, we have used mechanical infections in WT and DCL4.9i plants with either *in vitro* transcribed viroid RNA or total RNA from infected tissue and monitored PSTVd levels. We detected similar results to what we have observed before with agroinfiltration (S3 Fig).

**Fig 1:**
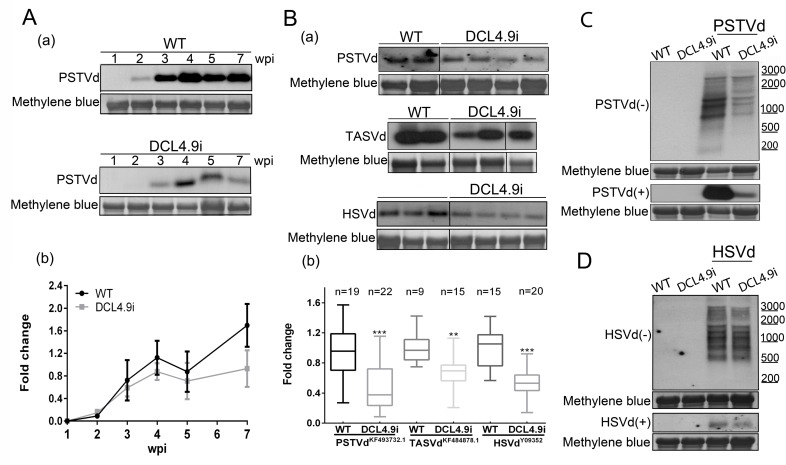
Further analysis of *Pospoviroidae* infections in DCL4i plants. (A) Time course of PSTVd infection in WT and DCL4.9i plants. Three plants were tested and a representative northern blot is presented. (B) Infections of WT and DCL4.9i plants with PSTVd^KF493732.1^, TASVd^KF484878.1^ (both at 5wpi) and HSVd^Y09352^ (3wpi). (C) Detection of (-) and (+) strand RNAs of PSTVd in WT and DCL4.9i plants and of HSVd in the same plants (D). In all northern blots, total RNA staining (methylene blue) was used as loading control. Quantification of northern blots was performed with Quantity One 4.4.1. Graphical representation and statistical analysis with Graphpad Prism 6 are presented in (b). ‘n’ corresponds to the number of individual plants tested. Results were analyzed with unpaired Student *t*-test, and the level of significance was set as p<0.01(**) and p<0.001 (***).

Finally, PSTVd levels were monitored in the course of infection in a 7 week period. We reasoned that if DCL4 suppression had an effect only at the initial events of the infection, viroid titer in DCL4i plants would eventually recover to WT levels. Three plants of each condition (WT and DCL4i) were infected with PSTVd and tissue of young leaves was collected at 1, 2, 3, 4, 5 and 7 wpi. As shown in Fig 1A, throughout infection, viroid levels are lower in DCL4i plants compared to WT plants. This suggests that DCL4 knock-down affects viroid accumulation throughout infection. Taken together these results imply that the observed reduction of viroid levels in the DCL4i plant line is sustained through the course of the infection and does not seem to be caused by an indirect effect of DCL4hp produced siRNAs or by problematic spatial PSTVd distribution.

### Decreased viroid levels in DCL4i plants infected with different *Pospiviroidae*

Next, we asked whether this phenomenon extends beyond PSTVd. Firstly, we tested whether the observed phenomenon in DCL4i plants is observed for another PSTVd strain additionally to the strain PSTVd^NB^. To this end, WT and DCL4i plants were infected with PSTVd^KF493732.1^ [51]), which was isolated from an infected tomato and differs from the NB strain in 20nt (S4 Fig). Then we extended our analysis to an additional member of the *Pospiviroidae* family of the same genus, *Tomato apical stunt viroid* (TASVd^KF484878.1^ [51]), an important viroid, recently (2014) added to the quarantine alert pest list of the European and Mediterranean plant protection organization (EPPO) [52]. This strain was isolated from a *Solanum jasminoides* plant, and present 75% identity to PSTVd^NB^ (S4 Fig). Both strains induce mild symptoms in *N*. *benthamiana*. Plants were mechanically infected and 5wpi upper leaves were collected and analyzed by northern blots, for viroid levels. As shown in Fig 1B, in both cases lower levels of viroid accumulation were observed in DCL4i plants. Additionally, we have infected by agroinfiltration WT and DCL4i plants with *Hop stunt viroid* (HSVd^Y09352^-Family: *Pospiviroid*, Genus *Hostuviroid* [53,54]), a viroid of the same family, but classified to a different genus. Three weeks post infection upper leaves were collected and reduced viroid levels in DCL4i plants were also observed (Fig 1B and S4 Fig).

### DCL4 suppression unlikely to act in *Pospoviroidae* replication

In an attempt to identify potential effects of DCL4 suppression in the replication of PSTVd we looked at (-) strand viroid RNAs, which are generally considered replication intermediates. Any over-accumulation or additional band of (-) strand viroid RNA, especially of the larger multiunit intermediates could indicate delay or other problems in specific steps of replication. Northern hybridization analysis of 3 weeks infected WT and DCL4i plants for the (-) strand RNA did not reveal significant differences in relative abundance of the bands corresponding to the (-) RNA species (Fig 1C). Similar findings were observed for HSVd (Fig 1D).

### Generation of plants with combined DCL decrease

**Fig 2:**
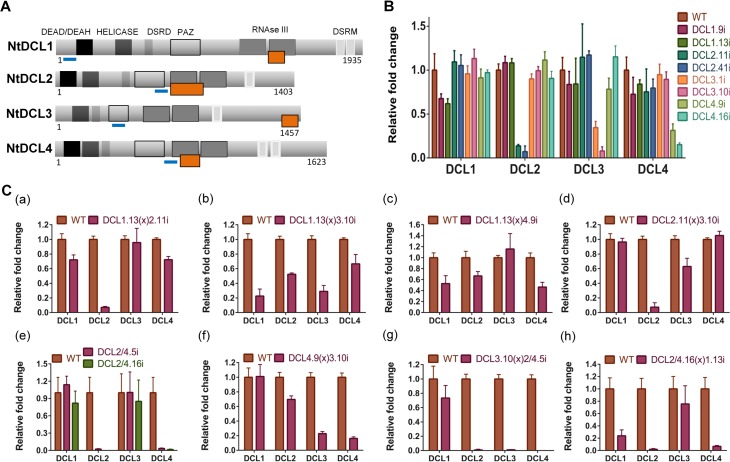
*N*. *benthamiana* DCLi plants have reduced levels of all targeted DCLs. (A) Schematic representation of *N*. *benthamiana* DCL proteins (based on information from [10]). Abbreviations stand for: DEAD-DEAH-DEAD box helicases, Helicase-Helicase C, DSRD-double-stranded RNA-binding domain, PAZ-Piwi Argonaute Zwille, RNAse III-Ribonuclease II, DSRM-double-stranded RNA-binding motif. The orange box represents the domain used to design the hairpins. The blue lines correspond to the selected fragment for qPCR analysis. (B) qPCR analysis of all DCLi single lines (n = 6). (C) qPCR analysis of F1 crosses line plants (n = 3–5) (a) DCL1.13(x)2.11i, (b) DCL1.13(x)3.10i, (c) DCL1.13(x)4.9i, (d) DCL2.11(x)3.10i, (e) DCL2/4.5i and DCL2/4.16i, (f) DCL4.9(x)3.10i, (g) DCL3.10(x)2/4.5i and (h) DCL2/4.16(x)1.13i.

An essential component of plants response to viruses is through the gene silencing mechanism. In order to investigate the role of gene silencing on PSTVd infectivity we used *N*. *benthamiana* plants suppressed for each and every Dicer and their combinations. Previously, we have shown the effect of individual DCL suppression on viroid accumulation [46]. The lines used were generated using RNAi-inducing hairpin constructs (Fig 2A) [46]. To avoid non specific effects due to the site of insertion of the T-DNA, two different lines were used for each DCL knock-down (for DCL1: 1.9i, 1.13i, for DCL2: 2.11i, 2.41i, for DCL3: 3.1i, 3.10i, for DCL4: 4.9i, 4.16i). In addition, we have generated a plant line that carries a hairpin against DCL2 and DCL4 simultaneously (DCL2/4i) [46]. We have performed quantitative PCR (qPCR) in these lines and determined mRNA levels of each DCL (Fig 2B). Each line successfully and specifically suppresses only the expected DCL. Homozygous DCLi plants where crossed to each other and F1 plants were used in this work, in order to avoid differences in the genetic background of the plants during infections. Relative levels of downregulation of each transcript in non infected plants were tested by qPCR and are presented in Fig 2C. DCL1.13(x)2.11i show a 28% decrease of DCL1 and 92.9% decrease of DCL2 transcripts. DCL1.13(x)3.10i has a downregulation of 77.4% of DCL1 mRNA and 71% of DCL3. DCL1.13(x)4.9i plants 47.3% and 53.5% of DCL1 and DCL4 transcripts respectively. DCL2.11(x)3.10i plants display a 92.6% and 37% reduction of DCL2 and DCL3 transcripts respectively. DCL2/4i plants present 97.7% and 96.5% reduction of the cognate transcripts. DCL4.9(x)3.10i plants have 77.2% reduction of DCL3 and 83.8% reduction of DCL4 compared to WT plants. Finally, triple knock-down plants DCL3.10(x)2/4.5i have decreased levels of around 98% for transcripts DCL2, DCL3 and DCL4 whereas DCL2/4.16(x)1.13i has a reduction of 76.2%, 98% and 93.2% for DCL1, DCL2 and DCL4 respectively (Fig 2C). It is to note that in a few cases other DCL proteins were mildly affected by the downregulation of two specific DCLs. Since no downregulation of the non targeted DCL were observed in the single DCLi lines, we argue that the observed effect is indirect.

**Fig 3:**
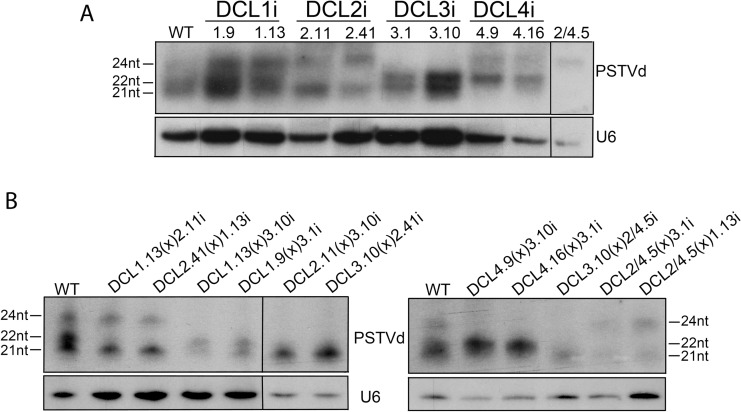
vd-siRNAs profiling in DCLi single or crossed plant lines. Small RNAs from 3 weeks infected *N*. *benthamiana* plants were analyzed in polyacrylamide gels and 21, 22 and 24nt of PSTVd were monitored. U6 was used as a loading control. In panel (A) DCLi single knock-down lines and in (B) DCLi F1 crosses. Quantification of small RNAs were made using Quantity One 4.4.1 shown in S2 Table.

Each DCL is responsible for the generation of siRNAs of a specific size class. As a consequence, suppression of specific DCLs should lead to suppression of the cognate siRNA species. Such reduction would indicate the efficient suppression of the DCL function. To this end DCLi plants were infected with PSTVd and 3wpi vd-siRNAs were investigated. As shown in Fig 3A (quantified results in S2 Table), PSTVd infected WT plants present three distinct vd-siRNAs classes of 21, 22 and 24nt long, with the two first being dominant. In DCL2i plants 22nt vd-siRNAs are not detected and 24nt are increased. In addition, in DCL2.41i line, a line with more efficient suppression of DCL2 than line DCL2.11i (Fig 2B), the increase of the 24nt population is even higher. This suggests that DCL3 products increase with the decrease of DCL2 indicating that DCL3 in WT conditions may be outcompeted for these substrates by DCL2. In DCL3i plants, the 24nt class are dimishished. In DCL4i plants, 21nt vd-siRNAs are strongly reduced and the 22nt class increased compared to WT. This could be an indication that DCL1 is not or marginally involved in the production of 21nt vd-siRNAs, since their production is significantly reduced in these RNAi plants. In DCL1.13(x)2.11i or DCL2.41(x)1.13i F1 plants no 22nt band is detected. In DCL1.13(x)3.10i and DCL1.9(x)3.1i no 24nt vd-siRNAs are detected, and in addition, there was no visible effect on the 21nt class. This is in accordance with a marginal (if any) role of DCL1 in the generation of vd-siRNAs under these conditions. When both DCL4 and DCL3 are suppressed (DCL4.9(x)3.10i and DCL4.16(x)3.1i F1 plants) both cognate vd-siRNA classes are bellow detection level. In the triple mutant DCL3(x)2/4i, it seems that the maternal origin of DCL3 is important, since when DCL3i (maternal) is crossed to DCL2/4i (paternal), only a faint band of 21nt is observed. In the reciprocal cross, when DCL2/4i is crossed to DCL3i, faint bands of 21nt and 24nt can be observed. Both cases differ from what is observed in DCL2/4i plants where only the 24nt class could be detected. Taken together, these results indicate that the produced crossed lines are efficiently and specifically downregulated for the cognate DCL(s) and this is mirrored by a specific alteration of the cognate vd-siRNA population. It should be noted that conclusions from this analysis cannot be drawn for DCL1, since a) the smallRNAs produced from the activity of this enzyme are of the same size as those of DCL4, and b) both DCL1i lines produced have a relatively low reduction of the targeted gene. This was not unexpected, since it was known from *A*. *thaliana* that strong *dcl1* suppression leads to embryo lethality [55].

### PSTVd infection does not significantly affect mRNA levels of RNAi components

**Fig 4:**
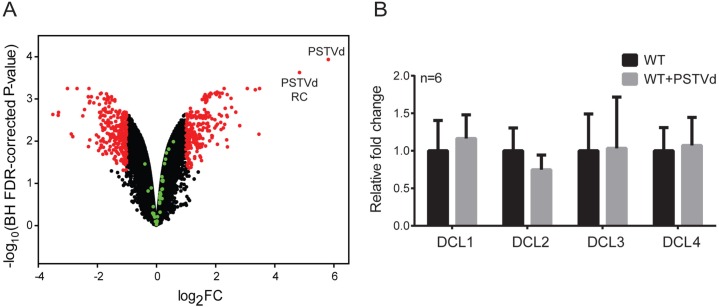
DCL levels upon PSTVd infection. (A) Volcano plot from microarray experiment with RNA from leaves of WT and PSTVd *N*. *benthamiana* infected plants (3wpi). *N*. *benthamiana* genes with no significant alteration of their expression level are indicated with black dots, while red dots represent genes with a significant higher or lower expression (fold change (FC) ≥ 2, Benjamini-Hochberg (BH) FDR-corrected P-value < 0.05) in PSTVd infected plants compared to WT plants. The 35 *N*. *benthamiana* RNAi components (described in [10]) are indicated with green dots. The PSTVd virus sequence and its reverse complement (RC) are both labeled (see micorarray design) (B) qPCR experiments of DCL transcripts upon infection. Two reference genes (L23, FBOX) were used for normalization. No significant differences were observed.

To investigate the interplay between the RNAi silencing machinery and PSTVd, we looked at the levels of principal components of RNAi following infection. We opted for a microarray analysis, in order to target all of the 35 different RNAi elements described for *N*. *benthamiana* [10]. To this end, we designed a custom genome-wide Agilent Gene Expression microarray (see material and methods for details). For genes related to the RNAi machinery, a total of 10 different probes for each transcript were designed [10]. In order to achieve statistical accuracy, replicates of WT and PSTVd infected plants were analyzed. As shown in a volcano plot (Fig 4A), no significant differences were found for any of the tested RNAi transcripts (green spots). The obtained values are presented in details in S3 Table. qPCR experiments for the four DCL transcripts in the same samples verified the microarray results, as no significant differences were observed (Fig 4B). Taken together, we have shown that PSTVd infection in our settings had no significant effect on the transcript levels of RNAi machinery.

### DCL4 suppression negatively affects PSTVd titer even when DCL1, DCL2 or DCL3 are also suppressed

**Fig 5:**
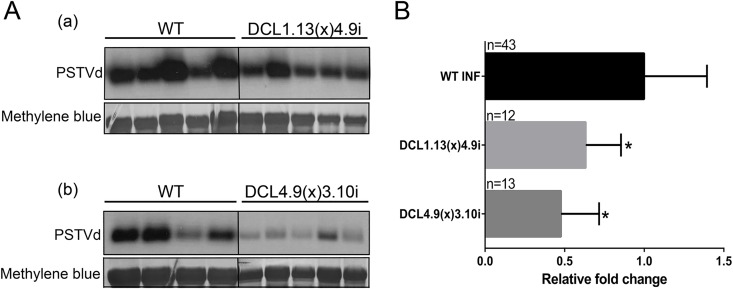
PSTVd infectivity in DCL1.13(x)4.9i and DCL4.9(x)3.10i plants. (A) Representative northern blots of DCL1.13(x)4.9i and DC4.9(x)3.10i PSTVd infected plants at 3wpi. Hybridizations were performed with DIG labeled (-) RNA strand of PSTVd, and total RNA staining (methylene blue) was used as internal loading control. (B) Graphical presentation of the infectivity quantification of PSTVd. Quantification was made using Quantity One 4.4.1 software. Student *t*-test was performed and significance level was set to p<0.05 (*). ‘n’ stands for the number of individual plants tested.

We previously showed that PSTVd is negatively affected upon DCL4 suppression. Here, we tested the effect of the combined suppression of DCL4 with each and every of the other three DCL enzymes on PSTVd infectivity. DCL1-DCL4 and DCL3-DCL4 knock-down plants infected with PSTVd for three weeks were analyzed by collecting RNA from young leaves and performing northern blots (Fig 5A). Quantified results from this analysis are presented in Fig 5B. As shown, the combined suppression of DCL1-DCL4 or DCL3-DCL4 genes led to a 36% and a 52.2% decrease of PSTVd titer respectively (Fig 5B). It is to note that in our previous work we have shown that simultaneous suppression of DCL2 and DCL4 also significantly reduced PSTVd levels [46]. These results show that DCL4 suppression has a negative impact on viroid titer even in combination with the suppression of any other individual DCL gene, demonstrating its important role in the PSTVd biological cycle.

### DCL2-DCL3 have a major role in the plant defense to PSTVd infection

**Fig 6:**
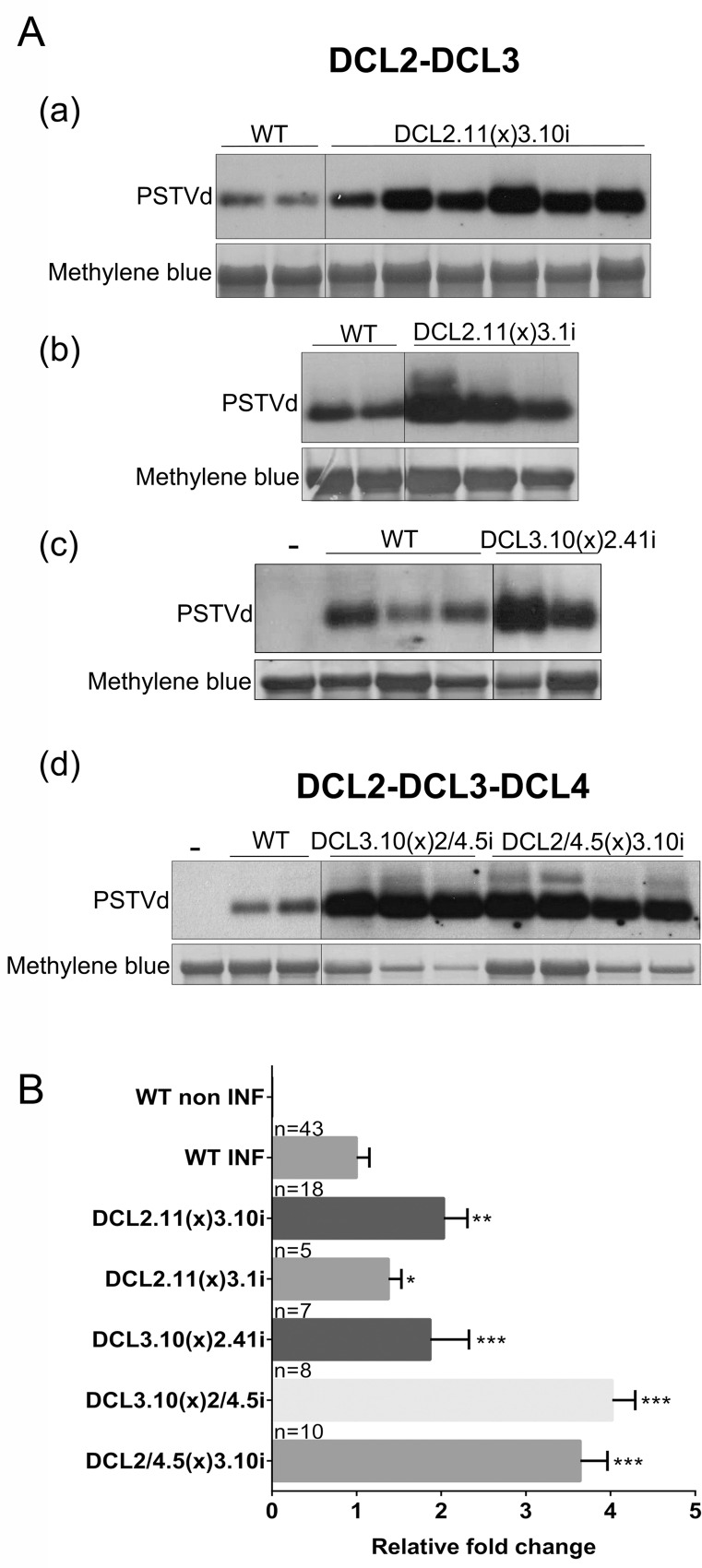
PSTVd infectivity in F1 DCLi crosses. PSTVd infected plants were analyzed at 3wpi. (A) Representative northern blots of (a) DCL2.11(x)3.10i, (b) DCL2.11(x)3.1i, (c) DCL3.10(x)2.41i and (d) DCL3.10(x)2/4.5i, DCL2/4.5(x)3.10i crosses. Total RNA staining (methylene blue) was used as loading control. Northern blots were quantified with Quantity One 4.4.1 software and are presented in (B). ‘n’, number of plants quantified. Student *t*-test was performed and significance levels were set as following: p<0.05 (*), p<0.01 (**) and p<0.001 (***).

Next, we examined the effect of suppression of multiple DCLs, other than DCL4, on PSTVd levels. As before, plants were infected by agroinfiltration with the PSTVd^NB^, and 3wpi young leaves were collected and examined in northern blots. Northern blots were quantified with appropriate software and results are presented in Fig 6 and S5 Fig. Infected plants DCL2.41(x)1.13i and DCL1.13(x)2.11i did not show any differences in PSTVd levels (S5 Fig). Experiments with knock-down plants of both DCL1 and DCL3 as well as with plants with decreased levels of DCL1, DCL2 and DCL4 were more complicated. Plants couldn’t be uniformly infected with PSTVd. Some plants showed increased levels and some could not be infected at all (S5 Fig). As described earlier [46], this phenomenon is also observed in DCL1i single knock-down plant lines and it is further discussed in the next section. As a result, experiments using DCL1i plants were considered as inconclusive.

The DCL2 and DCL3 knock-down plants had a strong positive effect on viroid accumulation. This was the case for all three different F1 DCL2i-DCL3i crosses tested in this work. DCL2.11i(x)3.10i showed on average a 2 fold increase of viroid levels, DCL2.11i(x)3.1i an almost 1.4 fold increase and finally DCL3.10i(x)2.41i a 1.9 fold increase (Fig 6). An important increase was also observed upon mechanical infection of PSTVd RNA (S6 Fig). This effect was even more pronounced when, in addition to DCL2 and DCL3, DCL4 was also suppressed. As shown in Fig 6Ad and 6B, a 4 and 3.6 fold increased PSTVd levels are observed in DCL3.10(x)2/4.5i and DCL2/4.5(x)3.10i F1 plants respectively.

Taken together, these results show that it is the combination of DCL2 and DCL3 together that is needed to defend against PSTVd efficiently. It seems that when both DCL2 and DCL3 are knocked down, a role for DCL4 in anti-viroid response can also be attributed. This indicates that although DCL4 most probably comes first to cleave the viroid, this pathway is not the most efficient suppressor of the viroid. In contrast, it is the DCL2 and DCL3 pathways which are more efficient in antiviroid defense but are put in the shade by the hierarchically first DCL4 processing. A model, integrating these findings is presented in Fig 8 and is discussed in the Discussion section.

**Fig 7:**
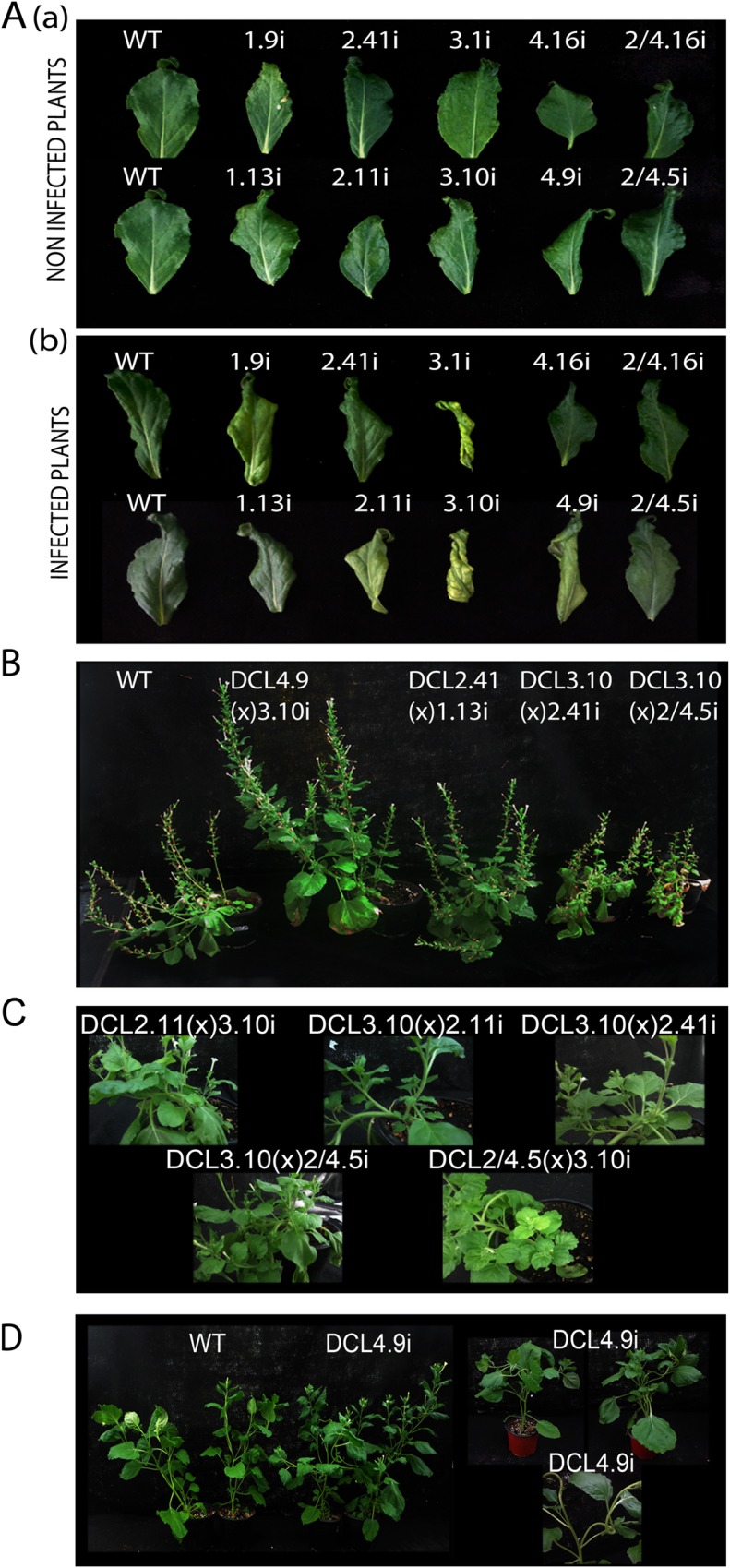
Phenotype of PSTVd infected F1 DCLi crosses. (A) Young leaves from non infected (a) and PSTVd infected (b) DCLi plants. (B) PSTVd infected DCLi crosses for 14wpi. (C) Increased branching observed in 5wpi PSTVd infected DCL3(x)2i, DCL2(x)3i and DCL3(x)2/4i plants. (D) Plants infected for 5 weeks with HSVd. Twisting of plants shoots can be observed.

In addition, we have found that these results are translated in differences observed in infected plant phenotypes. A significant ‘twisting’ of the upper leaves is observed in long time infected DCL3i lines (Fig 7A), which was not observed in WT plants, contributing to our suggestion of an important involvement of DCL3 in viroid defense. Furthermore, differentiation of PSTVd levels is mirrored by the observed severeness of symptoms in the various DCLi F1 crosses. As shown in Fig 7B, DCL4.9i(x)3.10i plants with decreased PSTVd titer, are less stunted than WT plants. At the opposite end, DCL3.10i(x)2.11i, DCL2.11(x)3.10i, DCL3.10i(x)2.41i and DCL3.10(x)2/4.5i F1 plants with increased PSTVd levels, show increased stunting and a ‘bushy’ effect, with increase of the branching and a decrease of the internode length, visible even at early weeks post infection (Fig 7C). An interesting phenotype is also observed in DCL4.9i plants infected with HSVd. They show twisted shoots not observed for the other viroids used here (Fig 7D). Taken together, these results show that the observed phenotypic effect of PSTVd infection follows closely viroid titers.

## Discussion

The complex relationship of viroids with the RNAi pathways has been puzzling researchers since the discovery of RNAi in 1998. The partially dsRNA nature of their genome, in combination with the lack of any silencing suppressor proteins, suggested that viroids could be a good target for RNAi resistance. This was supported a few years later by the finding that indeed vd-siRNAs are abundant in infected plants [4,36,37]. However, even though viroids seem to have evolved in such way that they can escape this fate leading to infection, the way they manage to do so remains unclear [39–42]. Nevertheless, different studies have implicated RNAi components in viroid infection [45–47]. In a previous study, we have shown that, at least, DCL2, DCL3 and DCL4 of *N*. *benthamiana* are involved in the production of cognate vd-siRNAs of specific size classes. In addition, we identified the importance of DCL4, since when DCL4 is suppressed, PSTVd titer and symptoms follow [46]. This effect is in contrast to what is usually observed during viral infections, where DCL4 acts as a major antiviral protein [13,19,20].

Here, we aimed to further characterize this phenomenon and to understand the role of individual DCL proteins during viroid infection. Our results demonstrated that a different PSTVd strain as well as two different members of the *Pospiviroidae* family (TASVd and HSVd) were also affected by DCL4 suppression in a similar way to the one shown before, suggesting that the observed phenomenon could be a general feature of this viroid family.

We estimated the effect of PSTVd infection on the expression levels of different RNAi components using a custom made gene-expression microarray followed by qPCR verification for the DCL genes. We did not find a significant effect of viroid infection on the expression of RNAi components. This is in contrast to what was shown previously for *Citrus Exocortis Viroid* (CEVd) where especially DCL4 levels increased as a result of CEVd infection [56]. This discrepancy may be due to differences in host species (*Solanum lycopersicum in* [56]*)*, the viroid used for the infection and/or the q-PCR normalization method, as only actin was used as a reference gene, a gene often affected by viral infections [57].

Further, we investigated effects of the simultaneous knock-down of more than one DCL protein, since functional redundancy between DCLs has been repeatedly reported [13,19,20,26–28]. DCLi *N*. *benthamiana* plants were crossed to each other in order to produce a set of plants suppressed for a different set of DCL proteins. Suppression through RNAi was effective even when all three DCL2, DCL3 and DCL4 were targeted simultaneously. This suggests that even small amounts of siRNAs produced by the remaining DCLs are enough to be loaded into AGO complexes and efficiently suppress endogenous sequences. The effects of DCL4 suppression were found epistatic to that of any other individual DCL (DCL1, DCL2 and DCL3) suppression, since whenever DCL4 was suppressed, the viroid accumulation was lower than in WT plants. However, when DCL2-DCL3 or DCL2-DCL3-DCL4 were simultaneously suppressed, PSTVd titer was found significantly increased. We have previously shown that infectivity in single DCL2i or DCL3i lines is not significantly affected at 3wpi [46]. Taken together, our present and previous observations suggest that it is the combined action of DCL2 and DCL3 that seem to be important in the plant response to PSTVd infection.

As far as we know, this is the first time that the combined effect of DCL2 and DCL3 against viroids or viruses has been observed. Analysis of viral infection for 3 (+) RNA viruses (CMV, TCV and TRV) in *dcl2dcl3* mutant *A*. *thaliana* plants showed no significant effect of their combined knockout on viral accumulation [19,20]. However, there are some indications that upon CMV infection, DCL3 can act to amplify the production of the 21nt viral siRNAs (vsRNAs) when DCL4 is suppressed. This indicates that DCL3 can eventually enhance the antiviral silencing by operating upstream of DCL4, although further elucidation of this effect is needed [58]. On the other hand, antiviral activity against DNA viruses such as *Cauliflower mosaic virus* (Family: *Caulimoiridae*, Genus: *Caulimovirus*) and *Cabbage leaf curl virus* (Family: *Geminiviridae*, Genus: *Begomovirus*) in *A*. *thaliana* relies on the action of all DCL proteins, although the activity of DCL3 is more pronounced, since an increased number of 24nt (compared to the abundance of this siRNA class in RNA viruses) is produced [13,59]. Additionally, an involvement of DCL1 has been proposed for CaMV and CaLCuV, which also differs from what it is observed for RNA viruses [13]. Both of these DNA viruses replicate in the nucleus as opposed to the majority of RNA viruses that replicate in the cytoplasm. Nevertheless, viroids differ from viruses. *Pospiviroidae* viroids replicate in the nucleus, thus it is tempting to speculate that the increased ‘need’ for DCL3 in host defense is due to this specific localization and partly resembles nuclear replicating viruses. This raises questions about where each DCL protein acts and why/how vd-siRNAs are mostly found in the cytoplasm [60].

We found strikingly contrasting effects of DCL2 and DCL4 in the plants antiviroid response. DCL2 is often described as acting in the shadow of DCL4 and is thought to have a role mainly in the absence of DCL4 to help in the restoration of its functions [19,20,61]. Both DCL2 and DCL4 have been known as important players in the antiviral response. Nevertheless, there have been increasing indications that DCL2 protein has a distinctive and possibly more effective role compared to DCL4. It has been shown that even though both DCL2 and DCL4 are necessary for gene silencing, DCL4 mainly act in the production of primary siRNAs, whereas DCL2 in the production of secondary siRNAs [17,18]. The authors propose that this dissimilarity is due to affinity differences of the dsRNA by DCL4 compared to DCL2 protein [18]. In addition, a role of DCL2 in RNA decay has been proposed [62]. Collectively, these works suggest that the DCL4-mediated pathway can serve as a decoy to antagonize the more destructive DCL2-mediated pathway, protecting either endogenous mRNAs from undesirable clearance or viral RNAs from degradation [18,62].

In the present work we investigated vd-siRNA in infected WT and DCLi plants. In WT, DCL4 and DCL2 produced vd-siRNAs (of 21 and 22nt class respectively), which are the main vd-siRNA classes produced upon viroid infection, followed by much smaller accumulation of 24nt DCL3 produced vd-siRNAs. Since deep sequencing studies have shown that vd-sRNAs of both polarities are found in more or less equal numbers [49,50], it is likely that these vd-siRNAs are either produced during viroid replication acting on the ds-vdRNA (as it has been proposed before [63]) or are cleavage products of RDR-produced dsRNA substrates. The latter is more likely given the abundance of vd-siRNAs of all classes in infected cells. RDR6 seems to be an important candidate in this process, since its involvement has been shown before. However, the implication of another RDR protein, such as RDR2 or even RDR1 cannot be ruled out [45]. A strong increase of the 22nt class is observed in infected plants knocked down for DCL4 or DCL3-DCL4, highlighting their possible importance correlated to DCL2 dicing activity, but also maybe more importantly to an anti-viroid AGO activity [30]. A milder yet important increase of the 24nt class is observed in DCL4i plants, suggesting a potential role for DCL3 in the absence of DCL4. Furthermore, lines DCL2.11i and DCL2.41i, which differ in the level of DCL2 suppression, showed an increase in the 24nt class and a decrease in 21nt class, proportionally to DCL2 suppression. In addition, previous observations have shown that 24nt production is increased with the course of the infection, eventually becoming as abundant as 21 and 22nt vd-siRNA [64]. Taken together, the above evidence suggests an important role of DCL3 in anti-viroid response.

**Fig 8:**
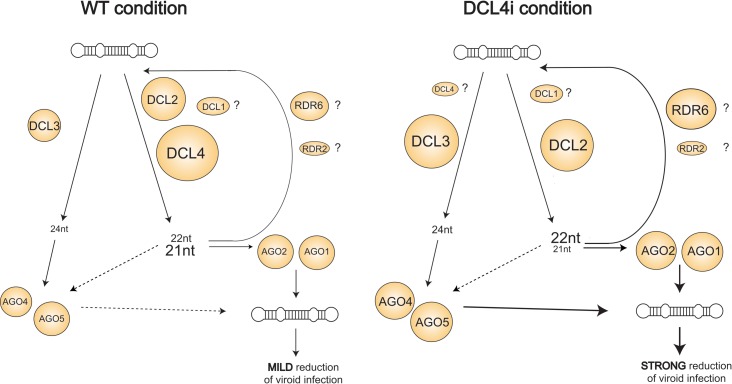
Proposed model of DCL involvement in PSTVd defense. In WT, PSTVd is targeted by at least three DCL enzymes. It is not clear whether DCL1 also contributes to this targeting. 21 and 22nt vd-siRNAs are loaded into AGO1 and AGO2 whereas 24nt into AGO4 and AGO5. These vd-siRNAs are probably redirected to target the PSTVd genome. In DCL4i condition, DCL4 is strongly suppressed, thus PSTVd genome is processed preferentially by a combination of DCL2-DCL3. The contribution of DCL1 in this action remains open. The majority of vd-siRNA produced at this condition are of 22nt class followed by 24nt class. These are probably uptaken by AGO1, AGO2, AGO4 and AGO5 and are targeting the PSTVd genome in a highly efficiently manner, resulting in a strong reduction of PSTVd infectivity.

The fate of the produced vd-siRNAs is probably affecting PSTVd levels. It has been shown that exogenous expression of *A*. *thaliana* AGO1, AGO2, AGO4 and AGO5 proteins in infected *N*. *benthamiana* plants decrease viroid levels. In addition, these AGOs bind 21 and 22nt vd-siRNAs. AGO4 and AGO5 additionally bind 24nt vd-siRNAs [47]. As described, a combination of DCL2-DCL3 knock-down leads to enhanced viroid infection whereas DCL4 reduction has the opposite effect. To integrate the results from this and other studies, we propose the following anti-viroid model (presented in Fig 8): In WT plants, PSTVd is targeted by DCL2, DCL3 and DCL4, producing mainly 21 and 22nt vd-siRNA, and a smaller portion of 24nt. The first two populations are preferentially loaded to AGO1 and AGO2, whereas 24nt vd-siRNAs to AGO4 and AGO5, driving plant anti-viroid defense probably through RDR proteins. The recent discovery of degradation products in PSTVd infected eggplants and to a less extent in infected *S*. *lycopersicum* and *N*. *benthamiana* supports this model [65]. The turnover is low compared to the very efficient replication rate and thus viroid infection is not significantly affected. Conversely, when DCL4 is suppressed, 22nt vd-siRNAs are loaded probably by AGO1/AGO2 and, together with the increased 24nt-mers loaded in AGO4, they are responsible for a more efficient targeting of the PSTVd leading to a strong decrease of PSTVd levels. This is further supported by the observation that DCL2 products stimulate RDR6 synthesis of secondary siRNAs [18]. In conclusion, the DCL4 pathway seems to be the least efficient against Pospiviroids, since its activity leads to a less effective suppression of the viroid and infection is efficient. In a sense, DCL4 is ‘protecting’ the viroid from the more ‘devastating’ effect of DCL2 and DCL3 processing.

For a long time, viroids ability to break plant resistance in spite of a functional silencing mechanism has been a conundrum. It has been suggested that vd-siRNA-mediated degradation was hindered due to viroids secondary structure [40] or through their localization to silencing free environments [66]. Although it is possible that viroid subcellular localization may aid viroid strategy in evading host defense, we believe that the present work highlights a novel important aspect in the survival strategy of viroids. Here we show that members of the *Pospiviroidae* family may have adapted in order to be primarily targeted and processed by DCL4, rather than the more hostile combination of DCL2 and/or DCL3. Even though they are processed by DCL4, the viroid overcomes this cellular response to produce an efficient infection. Understanding co-evolution of viroid-plant mechanisms of survival remains an interesting challenge for next studies on viroid pathogenesis.

## Materials and Methods

### Plants

*N*. *benthamiana* plants which contain hairpins to decrease endogenous DCL1, DCL2, DCL3 and DCL4 transcripts were described in [46]. A plant that expressed a hairpin for both DCL2 and DCL4 has also been created (DCL2/4.5i) [46]. Experiments were conducted in F6 to F10 generation. Combinations were created by crosses, and all experiments conducted in crossed plants were in F1 generation. We have produced the following crosses: DCL1.13(x)2.11i, DCL2.41(x)1.13i, DCL1.13(x)3.10i, DCL3.10(x)1.13i, DCL1.13(x)3.1i, DCL1.9(x)3.1i DCL1.13(x)4.9i, DCL2.11(x)3.10i, DCL3.10(x)2.41i, DCL4.9(x)3.10i, DCL4.9(x)3.1i DCL3.10(x)2/4.5i, DCL2/4.5(x)3.10i DCL2/4.5(x)3.1i and DCL2/4.16(x)1.13i.

### Infections

Two different type of infections were performed. For infections by agroinfiltration, plants at the stage of 5 leaves were agroinfiltrated with either *A*. *tumefaciens* GV3101 strain carrying an infectious PSTVd dimer (PSTVd^NB^-AJ634596) kindly provided by Dr. De Alba and Dr. Flores (Institute for Cellular and Molecular Plant Biology—IBMCP) or *A*. *tumefasciens* C58C1 strain containing plasmid pCdHSVd (HSVd^Y09352^) [54]. Samples were collected 3wpi.

For mechanical infection using carborundum (Prolabo, VWR), we either used infectious tissue from *N*. *benthamiana* containing TASVd^KF484878.1^ and PSTVd^KF493732.1^, provided by the Institute for Agricultural and Fisheries Research—ILVO, Belgium [51], or RNA. We have used 1μg of total RNA from PSTVd^NB^ infected WT *N*. *bentamiana* plant (S3 Fig). We have also used RNA from T3 *in vitro* transcription of plasmid *EcoR*I-pBKdNB provided by Dr. De Alba (S3A and S6 Figs). Used RNA quantities as well as time of infection are indicated in each experiment.

### RNA extraction and Northern

Young leaf samples were homogenized under liquid nitrogen and total RNA was extracted as previously described [46]. For large RNAs, five μg total RNA were separated in denaturing agarose gel (1.4% agarose, 0.7% formaldehyde) and transferred to 0.45μm nylon membrane (Whatman, GE healthcare). RNA (-) or (+) DIG labeled probes for PSTVd (DIG RNA labelling mix, Roche Diagnostics) were produced by either T7 transcription from a *Hind*III-pHa106 plasmid or by SP6 transcription from a *EcoR*I-pHa106 plasmid [67]. For the detection of HSVd viroid, DIG labeled in vitro transcription was produced either with T7 from *Kpn*1-pBdHSVd plasmid or with T3 from *Pst*I-pBdHSVd. Hybridization was performed over night at 65°C and CDP-star (Roche Diagnostics) was used for the detection according to the manufacturer instructions. For small RNAs, 20μg of total RNAs were migrated into 17% polyacrylamide gel (38:2) and transferred to 0.2μm nylon membrane (Whatman, GE healthcare). 100ng of a PSTVd PCR product produced by pHa106 plasmid with specific primers (S4 Table) was labeled with [α-32P]CTP using random priming reaction with Klenow (Minotech). Hybridization was performed at 50°C as described before [46]. For TASVd, a PCR product with specific primers (S4 Table) was produced and used exactly as for PSTVd with hybridization temperature at 65°C.

### Tissue print technique

Leaves of plants at 3wpi were cut and placed on a 0.45μm nylon membrane (Whatman, GE healthcare), with the upper side facing the membrane. A piece of Whatman paper was added at the other side of the leaf and, using a small rolling pin, leaves were pressed until the outline of the leaf was produced. The membrane was then used for DIG labeling as described above.

### Quantitative PCR

3μg of *DNAseI*-treated RNA were reverse transcribed with PrimeScript (Takara) using oligo-dT and random primers (Invitrogen). Kapa SYBR Fast qPCR kit was used to perform qPCR (Kapa Biosystems). All PCRs were carried out in a CFX CONNECT^TM^ apparatus (Biorad). Two reference genes (L23 and FBOX) were selected among different genes in order to have a p<0.05 using NormFinder and BestKeeper algorithm [68,69]. Analysis was performed using either qBASE or Pfaffl algorithm. Annealing temperatures as well as used primers are described in S4 Table.

### Microarray construction

A custom Sureprint genome-wide G3 Gene Expression 4×180k microarray (Agilent design ID 074128) was designed using the Agilent eArray platform (Agilent Technologies) based on the *N*. *benthamiana* genome annotation (“Niben101”, 57140 transcripts, version from March 6, 2015, available at ftp://ftp.solgenomics.net/genomes/Nicotiana_benthamiana/annotation/Niben101/) and sequences of *N*. *benthamiana* RNA silencing (RNAi) genes reported by [10] (35 genes, available at http://sefapps02.qut.edu.au/benWeb/subpages/downloads.php). For *N*. *benthamiana* RNAi genes (35 genes) and *N*. *benthamiana* transcripts having a BLASTx hit (E-value < E-10) with either *A*. *thaliana* RNAi protein sequences [9] or amino acid translations of *N*. *benthamiana* RNAi genes (407 transcripts in total), probe design aimed for four probes of 60 nt per gene-transcript with parameters set to “best probe methodology” and “3’bias” and six probes of 60 nt per gene-transcript with parameters set to “best probe distribution” and “without 3’ bias”. For the remainder of the *N*. *benthamiana* transcripts, probe design aimed for three probes of 60 nt per gene with parameters set to “best probe distribution” and “3’bias”. In the microarray design, we also included 3 probes (“3’bias”, “best probe distribution”) for the PSTVd genome sequence and 3 probes for its reverse complement. In total 173,491 probes were created and 98.5% of all *N*. *benthamiana* transcripts-genes (56310 sequences) had at least three probes per transcript-gene, while only 263 transcripts had no probes. Finally, we also designed 8 probes per transcript (“3’bias”, “best probe methodology”, (S5 Table) for 38 *N*. *benthamiana* housekeeping transcripts. These probes were randomly distributed in 9 copies per array and were used to measure intra-array reproducibility (“replicate non-control probe group”). The array design was submitted to the National Center for Biotechnology Information (NCBI) under the Gene Expression Omnibus (GEO)-platform format (GPL21946).

### Target preparation, microarray hybridization, and analysis

*N*. *benthamiana* WT plants infected or not with PSTVd for three weeks were used. Four biologically replicated RNA samples were obtained. Large RNAs were extracted as described earlier. Quantity and integrity was measured using an Agilent TapeStation system. RNA samples were labelled with cyanine dyes following the Low Input Quick Amp Labeling Kit (Agilent Technologies), with 100 ng of total RNA as starting material. RNA samples from uninfected *N*. *benthamiana* plants were labeled with cy3, while cy5-labelling was performed for RNA samples of PSTVd infected plants. Samples were hybridized to a custom-made Sureprint G3 4x180K array (Agilent Technologies (see above)) following the standard procedure of the Gene Expression Hybridization Kit (Agilent Technologies). The following hybridization experiment was performed (number of biological replicates is given between brackets): Cy5 labeled cRNA from WT plants infected with PSTVd versus Cy3 labeled RNA from WT plants (4). After washing procedures (Gene Expression Wash Buffer kit (Agilent Technologies)), the 4x180k slide was scanned by an Agilent high-resolution microarray scanner (Agilent Technologies), raw data was extracted from the 4x180k slide using the GE2_107_Sep09 protocol of the Agilent Feature Extraction Software and subsequently transferred to limma for further processing and statistical analysis [70]. Based on the arrayQualityMetrics report [71] one array (array 1_4) was considered as outlier, and data from this array was excluded from further analysis. Before creating the microarray (MA) files, data were processed by background correction using the ‘normexp’ method at offset 50. Microarray data (MA) were then normalized within and between arrays by loess and Aquantile, respectively [72]. Using the normalized MA-object, differential expression was assessed by an empirical Bayes approach with cut-offs for the Benjamini–Hochberg FDR-corrected P-values and log2-converted FC [log2(FC)] at 0.05 and 1 respectively [73]. *N*. *benthamiana* gene expression data have been uploaded to the Gene Expression Omnibus with accession number GSE81923.

### *In silico* analysis of the DCL4.9i hairpin construct

Artificial siRNA sequences were generated from the DCL4.9i hairpin sequence using a custom python script and a 21-nucleotide sliding window. These siRNA sequences (S1 Table) were used as query in a blastn search against the PSTVd genome with following blastn parameters (word_size 7, penalty -1, gapopen 1, gapextend 2, evalue 1000) as in [74].

### Software

For the analysis of the northern blots, Quantity One 4.4.1 (Biorad) was used. Values were calculated and compared first to methylene blue values and then to the WT values. Statistical analysis was performed using GraphPad Prism 6 software (GraphPad software Inc).

## Supporting Information

S1 Fig siRNAs from the hairpin do not directly affect PSTVd levels. (A) Representative image of a PSTVd infected leaf, agroinfiltrated in one half part with GFP and the other half with pk7-DCL4hp [46]. (B) PCR for the loop of the hairpin (spacer). A F-BOX gene was used as PCR internal control (C) Northern blot for PSTVd levels of either GFP or DCL4hp agroinfiltrated leaves. Total RNA staining (methylene blue) was used as control. (D) Quantification of the northern blot of n = 14 different leaves. No significant difference in PSTVd levels between GFP and DCL4hp agroinfiltrated parts is observed.
(TIF)

S2 Fig PSTVd distribution in WT and DCL4i *N*. *benthamiana* tissues. Tissue prints of all infected leaves in WT and DCL4i plants. Hybridization was performed with DIG labeled (-) RNA.
(TIF)

S3 Fig Mechanical infections of WT and DCL4.9i plants. (A) Northern blot of *N*. *benthamiana* plants 5 wpi with 500ng of *in vitro* transcript of PSTVd^NB^ strain. Total RNA (stained with methylene blue) was used as loading control. (B) Northern blot of *N*. *benthamiana* plants infected for 4 weeks with PSTVd^NB^. Infection were performed using 1μg total RNA from 7wpi infected tissue. Total RNA staining (methylene blue) was used as loading control. Lane 2 corresponds to a non infected WT plant (-). (b) Quantification of Northern blots using Quantity One 4.4.1. ‘n’ corresponds to the number of plants tested. Student *t*-test was performed with significant level at p<0.05 (*).
(TIF)

S4 Fig Alignment of *Pospiviroidae* used in this study. (A) PSTVd^KF493732.1^, (B) TASVd^KF484878.1^ and (C) HSVd^Y09352^ were aligned to PSTVd^NB^ using the online MUSCLE software [75].
(TIF)

S5 Fig PSTVd infectivity levels in DCL1(x)DCL2i, DCL1(x)DCL3i and DCL1(x)DCL2/4i F1 crosses. Representative northern blots of F1 crosses (A) DCL2.41(x)1.13i, DCL1.13(x)2.11i, (B) DCL1.13(x)3.10i, (C) DCL3.10(x)1.13i, (D) DCL1.13(x)3.1i and (E) DCL2/4.16(x)1.13i. Hybridizations were performed with DIG labeled (-) PSTVd RNA. Total RNA staining (methylene blue) was used as loading control.
(TIF)

S6 Fig Mechanical infections of WT and DCL3.10(x)2.41i plants. (A) PCR from *N*. *benthamiana* PSTVd^NB^ infected plants at 4wpi. Mechanical infections were produced with RNA transcribed from *Eco*RI-pBSK-dNB plasmid using 1μg per leaf according to [76]. L23 was used as an internal control for PCR. (B) Northern blot of the same plants at 7wpi. Total RNA staining (methylene blue) was used as loading control.
(TIF)

S1 Table *In silico* predicted siRNAs produced by DCL4 hairpin. (XLSX)

S2 Table Quantification of PSTVd siRNAs of Fig 3. Quantifications were performed using software Quantity One 4.4.1. For the detection of 21nt and 22nt- mers, since they do not fully separate, we have arbitrary split the area showing signal from both the 21 and 22nt bands into two equal parts and taken the upper as representative of 22nt signal and the lower of 21nt siRNA signal. Values were normalized to U6 and compared to the cognate signals from the WT infected plants
(XLSX)

S3 Table Log2FC and Benjamini-Hochberg FDR-corrected P-value of *N*. *benthamiana* RNAi genes in WT plants infected with PSTVd compared to WT plants. (XLSX)

S4 Table Primers used in this study. (DOCX)

S5 Table Accession IDs of *N*. *benthamiana* transcripts used for measurement of intra-array reproducibility. (XLSX)
