# Supplementary figures and images for: Combined Activity of DCL2 and DCL3 Is Crucial in the Defense against Potato Spindle Tuber Viroid
Source: PLoS Pathog. 2016 Oct 12;12(10):e1005936. doi: 10.1371/journal.ppat.1005936 (PMC5061435; doi:10.1371/journal.ppat.1005936)

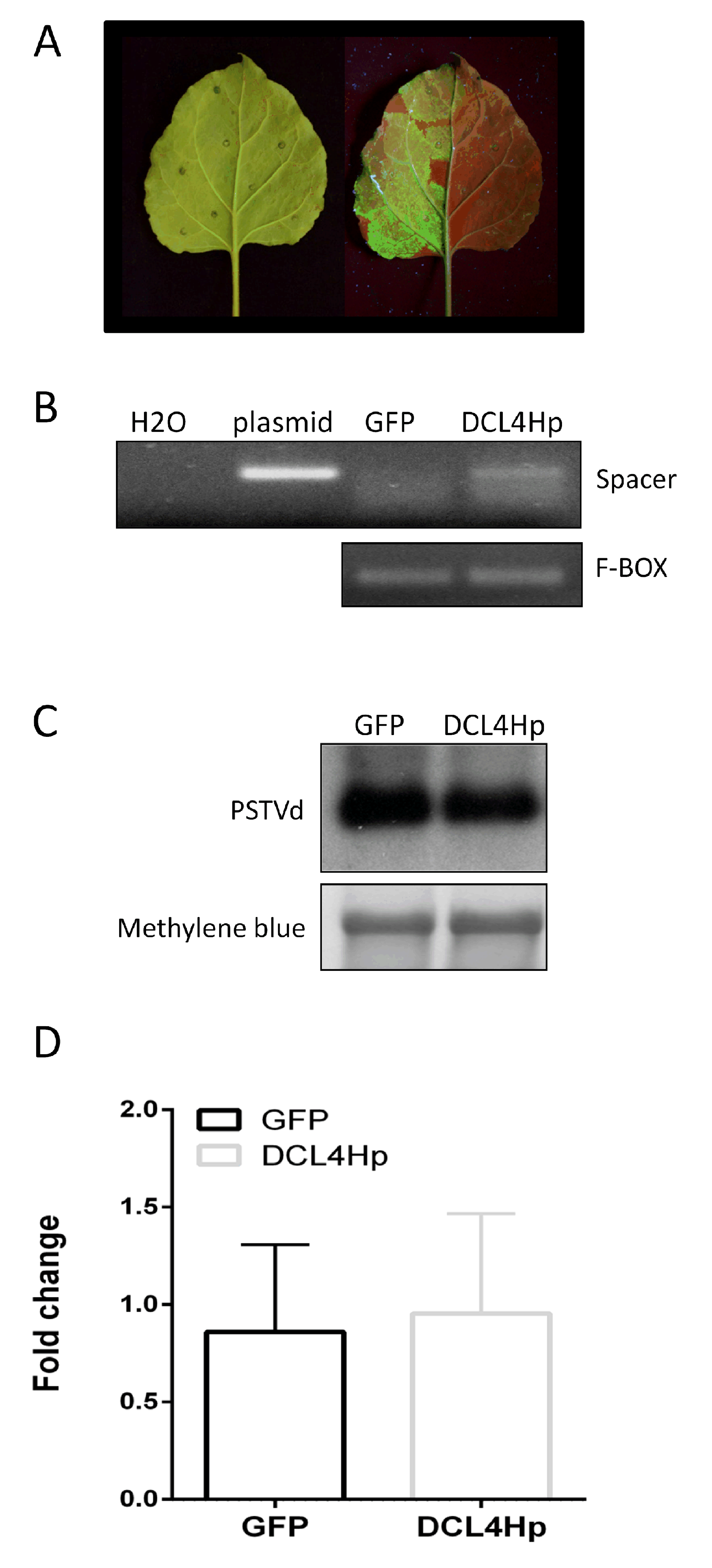

Supplement: S1 Fig — (A) Representative image of a PSTVd infected leaf, agroinfiltrated in one half part with GFP and the other half with pk7-DCL4hp [46]. (B) PCR for the loop of the hairpin (spacer). A F-BOX gene was used as PCR internal control (C) Northern blot for PSTVd levels of either GFP or DCL4hp agroinfiltrated leaves. Total RNA staining (methylene blue) was used as control. (D) Quantification of the northern blot of n = 14 different leaves. No significant difference in PSTVd levels between GFP and DCL4hp agroinfiltrated parts is observed. (TIF) [file ppat.1005936.s001.tif]

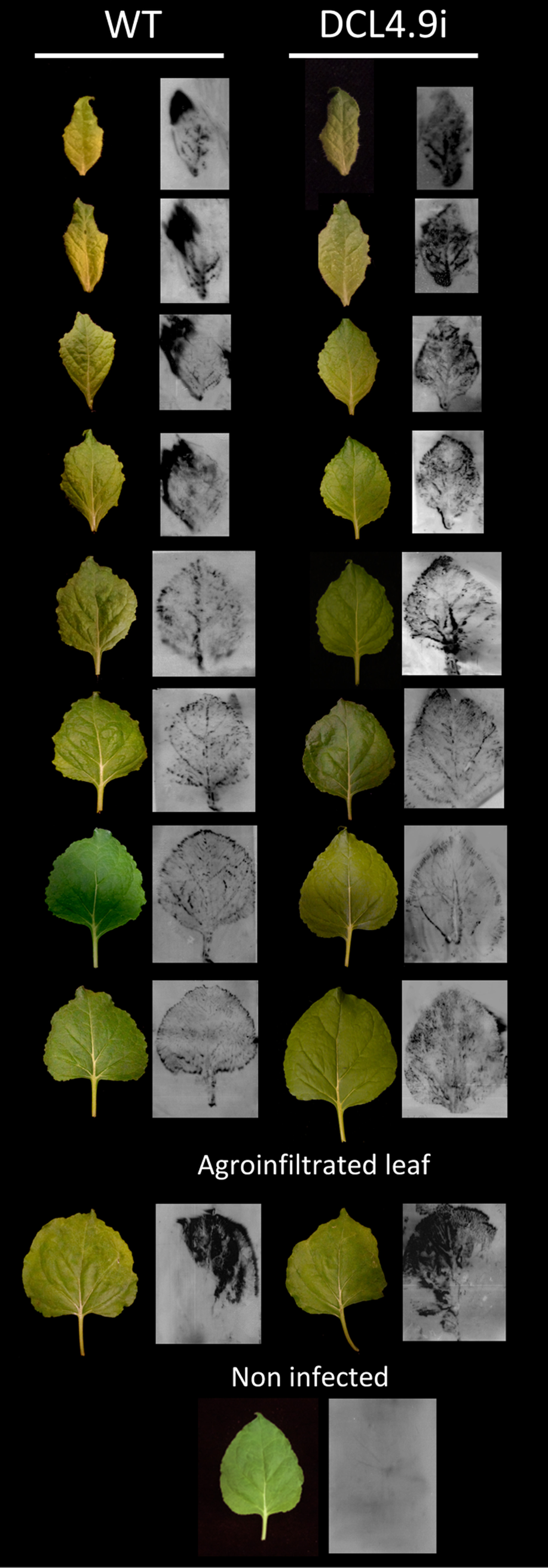

Supplement: S2 Fig — Tissue prints of all infected leaves in WT and DCL4i plants. Hybridization was performed with DIG labeled (-) RNA. (TIF) [file ppat.1005936.s002.tif]

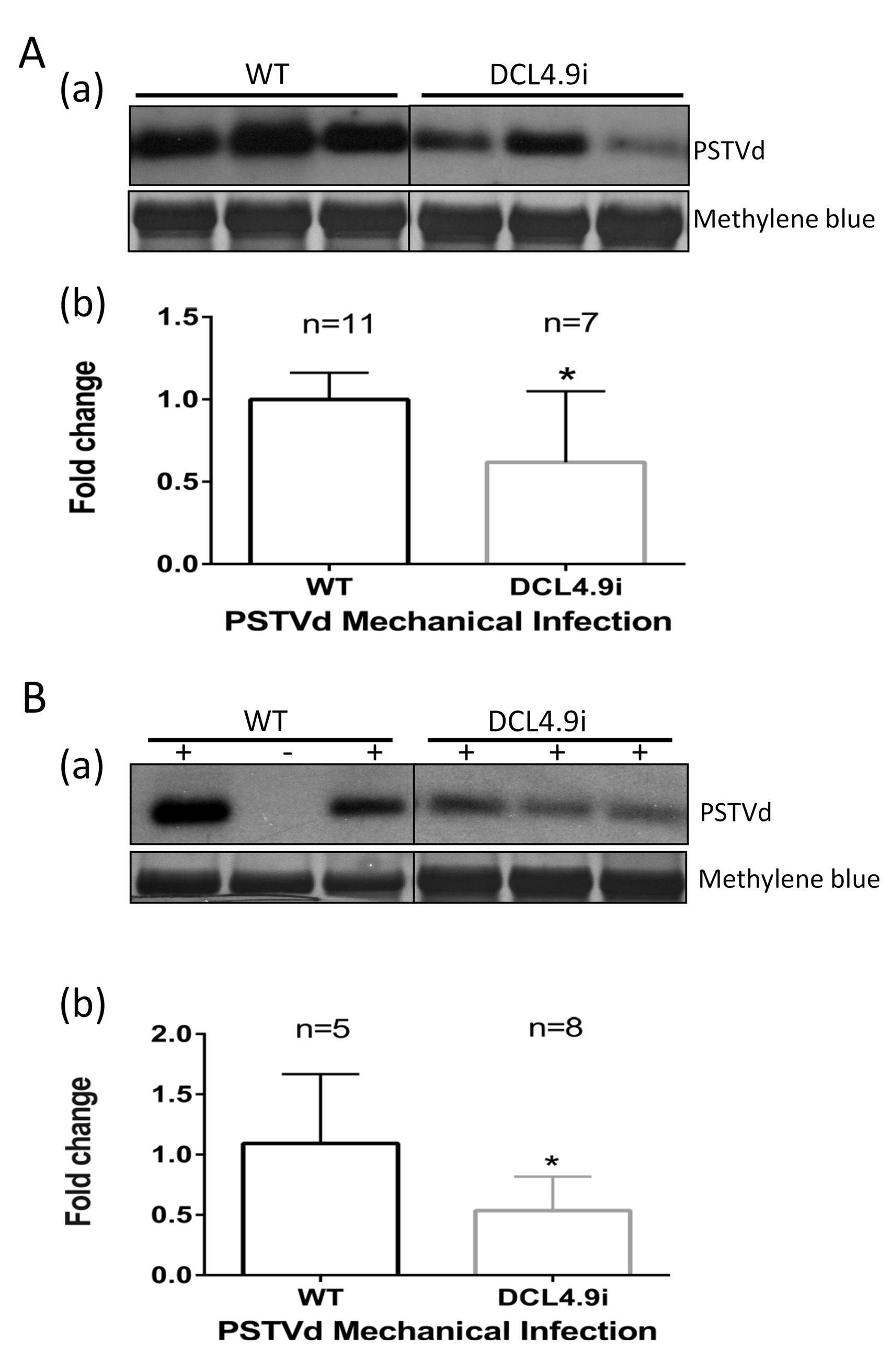

Supplement: S3 Fig — (A) Northern blot of N. benthamiana plants 5 wpi with 500ng of in vitro transcript of PSTVdNB strain. Total RNA (stained with methylene blue) was used as loading control. (B) Northern blot of N. benthamiana plants infected for 4 weeks with PSTVdNB. Infection were performed using 1μg total RNA from 7wpi infected tissue. Total RNA staining (methylene blue) was used as loading control. Lane 2 corresponds to a non infected WT plant (-). (b) Quantification of Northern blots using Quantity One 4.4.1. ‘n’ corresponds to the number of plants tested. Student t-test was performed with significant level at p<0.05 (*). (TIF) [file ppat.1005936.s003.tif]

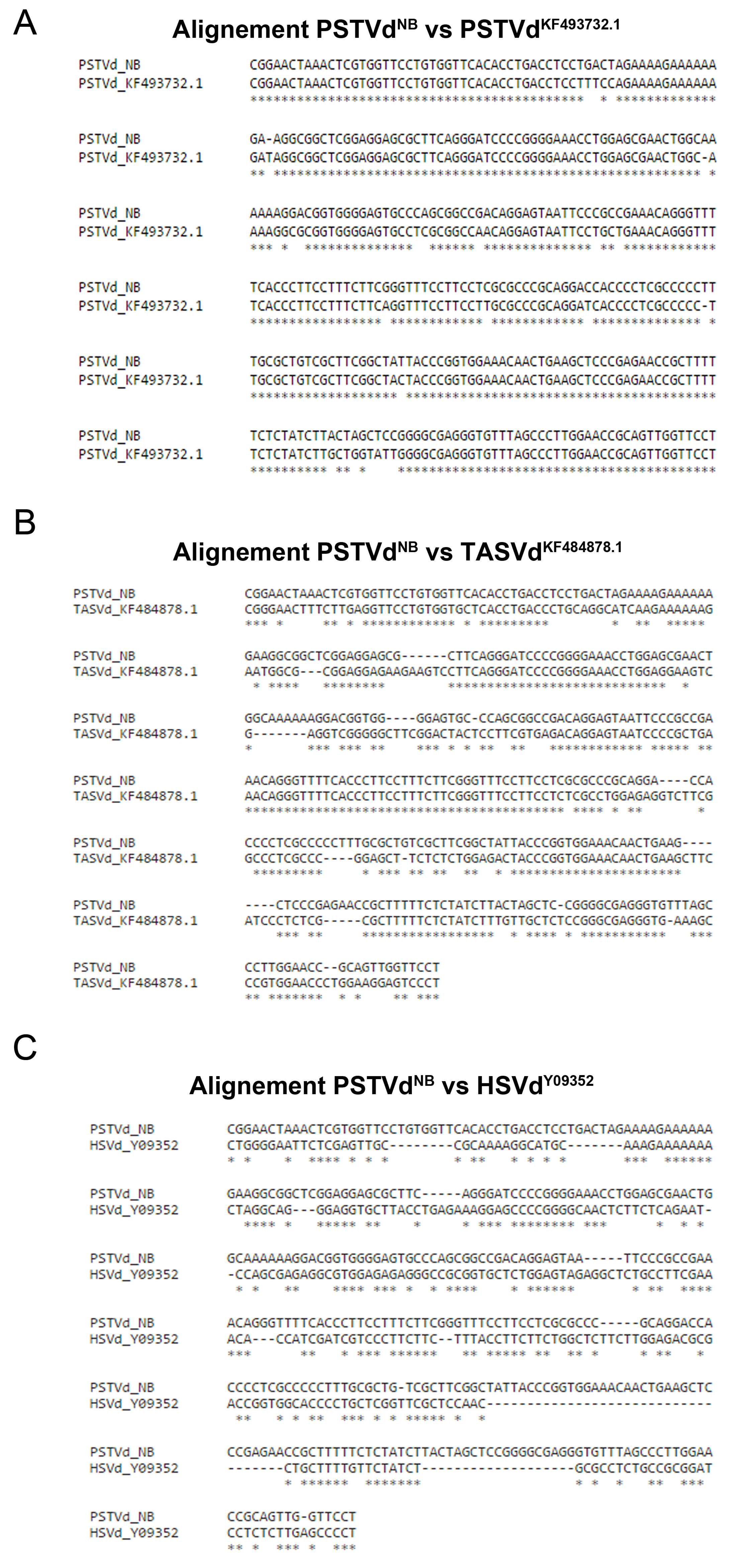

Supplement: S4 Fig — (A) PSTVdKF493732.1, (B) TASVdKF484878.1 and (C) HSVdY09352 were aligned to PSTVdNB using the online MUSCLE software [75]. (TIF) [file ppat.1005936.s004.tif]

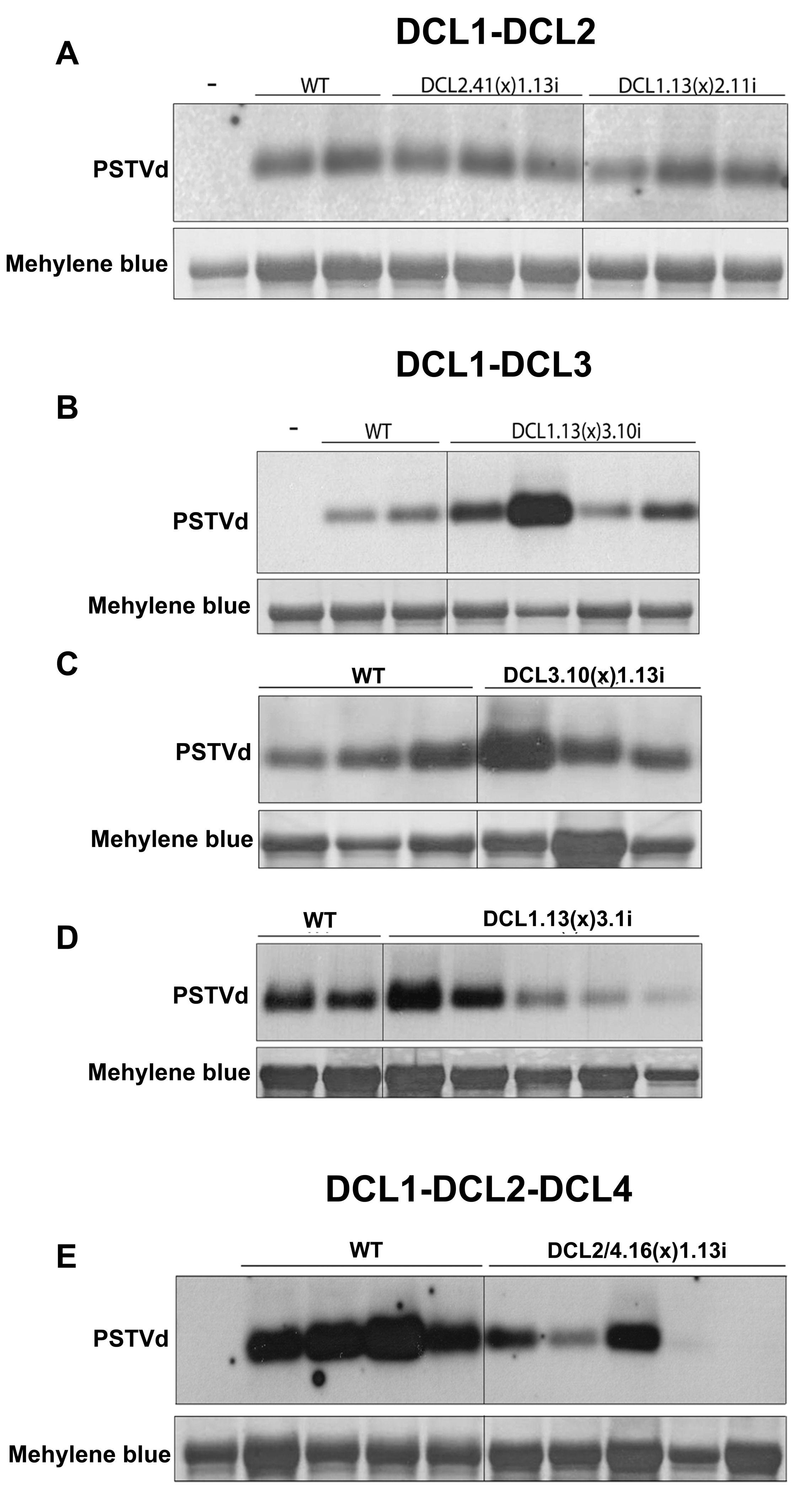

Supplement: S5 Fig — Representative northern blots of F1 crosses (A) DCL2.41(x)1.13i, DCL1.13(x)2.11i, (B) DCL1.13(x)3.10i, (C) DCL3.10(x)1.13i, (D) DCL1.13(x)3.1i and (E) DCL2/4.16(x)1.13i. Hybridizations were performed with DIG labeled (-) PSTVd RNA. Total RNA staining (methylene blue) was used as loading control. (TIF) [file ppat.1005936.s005.tif]

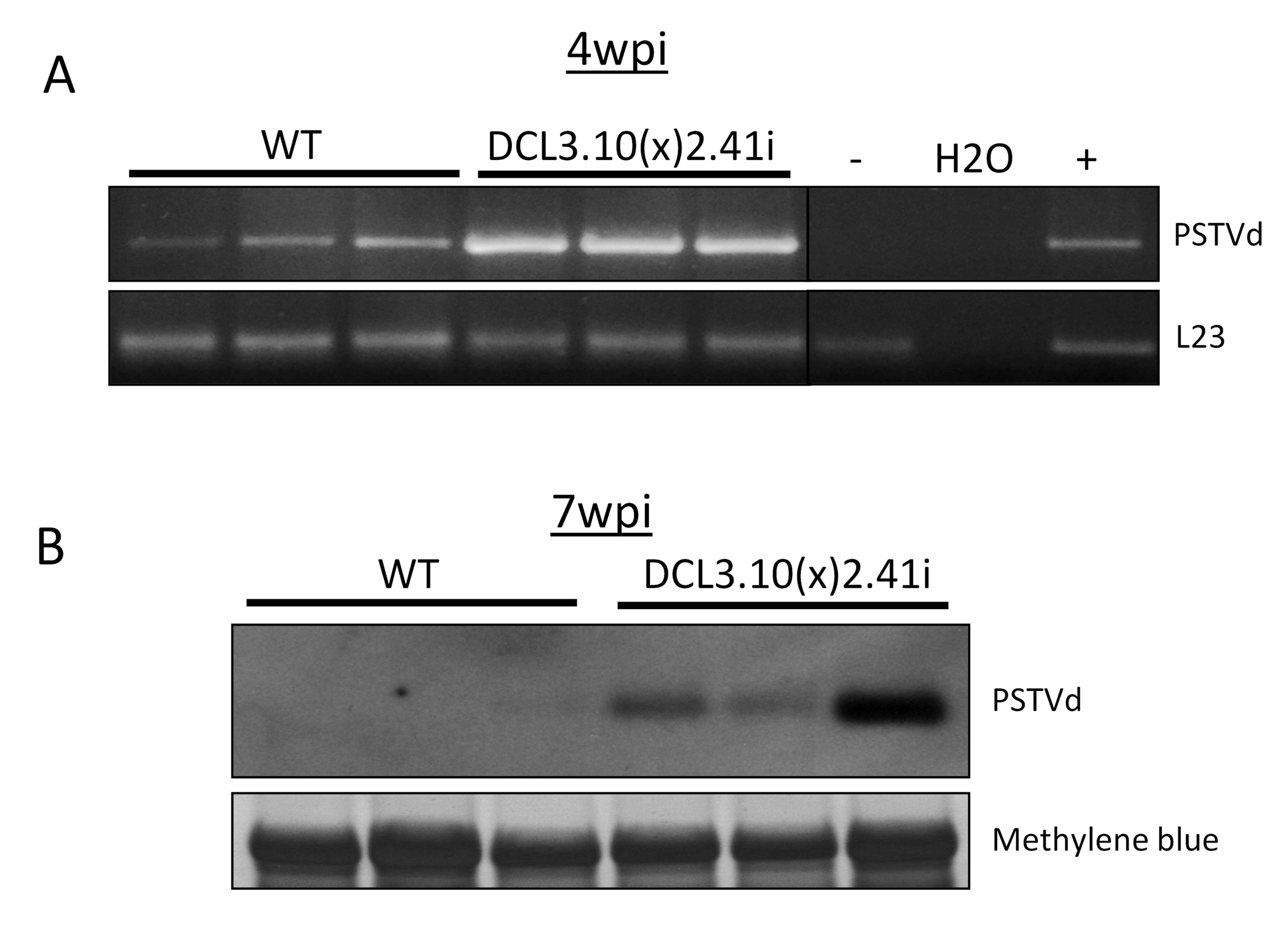

Supplement: S6 Fig — (A) PCR from N. benthamiana PSTVdNB infected plants at 4wpi. Mechanical infections were produced with RNA transcribed from EcoRI-pBSK-dNB plasmid using 1μg per leaf according to [76]. L23 was used as an internal control for PCR. (B) Northern blot of the same plants at 7wpi. Total RNA staining (methylene blue) was used as loading control. (TIF) [file ppat.1005936.s006.tif]
