# Supplementary material for: Combined Activity of DCL2 and DCL3 Is Crucial in the Defense against Potato Spindle Tuber Viroid
Source: PLoS Pathog. 2016 Oct 12;12(10):e1005936. doi: 10.1371/journal.ppat.1005936 (PMC5061435; doi:10.1371/journal.ppat.1005936)
Supplement: S4 Table — (DOCX) [file ppat.1005936.s010.docx]

**S4_Table:** Primers used in this study

| **Name** | **Sequence (5’-3’)** | **Purpose** | **Used T^o^C** | **Reference (If any)** |
| --- | --- | --- | --- | --- |
| **qNbDCL1-F** | AAAAGAATGAGATGGTATTTCGG | qPCR | 55^o^C | [1] |
| **qNbDCL1-R** | TTCTTTCTGGCATGCTCAA | qPCR | 55^o^C | [1] |
| **qNbDCL2-F** | GAAGAACCACTTCTTAGGGGAAA | qPCR | 59^o^C | [1] |
| **qNbDCL2-R** | GGCCATAACAAGGACTCAA | qPCR | 59^o^C | [1] |
| **qNbDCL3-F** | AGGACTGCAGCGTTTATGGT | qPCR | 56^o^C | Present work |
| **qNbDCL3-R** | GGCCACAACAACCATGTCCT | qPCR | 56^o^C | Present work |
| **DCL4_For b** | GCACTTAACTACAGAGAAATGCAATG | qPCR | 60^o^C | [2] |
| **DCL4_Rev b** | ACAATGTTTGAGCGCCTTCT | qPCR | 60^o^C | [2] |
| **F-BOX-F** | GGCACTCACAAACGTCTATTTC | qPCR/PCR | 62^o^C | [3] |
| **F-BOX-R** | ACCTGGGAGGCATCCTGCTTAT | qPCR/PCR | 62^o^C | [3] |
| **L23-F** | AAGGATGCCGTGAAGAAGATGT | qPCR/PCR | 60^o^C | [3] |
| **L23-R** | GCATCGTAGTCAGGAGTCAACC | qPCR/PCR | 60^o^C | [3] |
| **PSTVd-For** | AACTCGAGCGGAACTAAACTCGTGGTT | Probe/PCR | 45^o^C(probe) 54^o^C (PCR) | Present work |
| **PSTVd-Rev** | AAGAATGAGGAACCAACTGCGGTTCC | Probe/PCR | 45^o^C(probe) 54^o^C (PCR) | Present work |
| **TASVd -For** | AAAAAGAATGGCGCGGAGGA | probe | 45^o^C (probe) 60 ^o^C (PCR) | Present work |
| **TASVd-Rev** | AGAGAAAAAGCGCGAGAGGG | probe | 45^o^C (probe) 60 ^o^C (PCR) | Present work |
| **pk7-Spacer-For** | ACCCACTAAGCGTGACCAGA | PCR | 60^o^C | Present work |
| **pk7-Spacer-Rev** | CGGGATCAGAAGCAACCTCA | PCR | 60^o^C | Present work |

**Supplemental Bibliography for S4_Table**

1. Dadami E, Boutla A, Vrettos N, Tzortzakaki S, Karakasilioti I, Kalantidis K. DICER-LIKE 4 but not DICER-LIKE 2 may have a positive effect on potato spindle tuber viroid accumulation in Nicotiana benthamiana. Mol Plant. 2013;6: 232–234.

2. Kotakis C, Vrettos N, Kotsis D, Tsagris M, Kotzabasis K, Kalantidis K. Light intensity affects RNA silencing of a transgene in Nicotiana benthamiana plants. BMC Plant Biol. 2010;10: 220.

3. Liu D, Shi L, Han C, Yu J, Li D, Zhang Y. Validation of Reference Genes for Gene Expression Studies in Virus-Infected Nicotiana benthamiana Using Quantitative Real-Time PCR. PLoS One. 2012;7: e46451.
